# Omics are Getting Us Closer to Understanding IgA Nephropathy

**DOI:** 10.1007/s00005-023-00677-w

**Published:** 2023-04-15

**Authors:** Krzysztof Mucha, Michał Pac, Leszek Pączek

**Affiliations:** 1 Department of Immunology, Transplantology and Internal Diseases, Medical University of Warsaw, Warsaw, Poland; 2 Institute of Biochemistry and Biophysics, Polish Academy of Sciences, Warsaw, Poland

**Keywords:** IgA nephropathy, Immune system, Epigenomics, Genomics, Proteomics, Transcriptomics

## Abstract

During the last decade, thanks to omics technologies, new light has been shed on the pathogenesis of many diseases. Genomics, epigenomics, transcriptomics, and proteomics have helped to provide a better understanding of the origin and heterogeneity of several diseases. However, the risk factors for most autoimmune diseases remain unknown. The successes and pitfalls of omics have also been observed in nephrology, including immunoglobulin A nephropathy (IgAN), the most common form of glomerulonephritis and a principal cause of end-stage renal disease worldwide. Unfortunately, the immense progress in basic research has not yet been followed by the satisfactory development of a targeted treatment. Although, most omics studies describe changes in the immune system, there is still insufficient data to apply their results in the constantly evolving multi-hit pathogenesis model and thus do to provide a complete picture of the disease. Here, we describe recent findings regarding the pathophysiology of IgAN and link omics studies with immune system dysregulation. This review provides insights into specific IgAN markers, which may lead to the identification of potential targets for personalised treatment in the future.

## Introduction

Immunoglobulin A nephropathy (IgAN) is the most common form of primary glomerulonephritis worldwide (Wyatt and Julian 2013). Progression to end-stage renal disease (ESRD) within 30 years occurs in up to 50% of cases (Moriyama et al. 2014). IgAN typically affects people between the second and third decade of life and is the most common cause of ESRD in young adults (Penfold et al. 2018). The prevalence of IgAN has a West-to-East gradient with the highest incidence rates in Japan and China, which might be the result of local environmental pressures (Kiryluk et al. 2012).

The clinical manifestation of IgAN is highly variable and can range from asymptomatic haematuria and proteinuria to gross haematuria with rapid progression to renal insufficiency.

Although more than a half century has passed since IgAN was first described by Berger and Hinglais (1968), a comprehensive understanding of its pathogenesis remains unknown. The widely accepted, albeit still developing multi-hit pathogenesis model, describes IgAN as a systemic disease that consists of four steps: the production of galactose-deficient IgA_1_ (Gd-IgA_1_; hit 1), the production of autoantibodies recognizing Gd-IgA_1_ (hit 2), immune complex formation (hit 3), and mesangial deposition (hit 4) (Suzuki et al. 2011). The gold standard for IgAN diagnosis is renal biopsy. The findings most commonly observed by immunofluorescence microscopy are mesangial deposits of IgA (consisting of poorly O-glycosylated polymeric IgA_1_ with predominance of the lambda light chain), often accompanied by IgM, IgG, or complement C3 component (Roberts et al. 2009). A systemic form of IgA-related disease, Henoch-Schönlein purpura nephritis characterised by purpura, arthralgia, abdominal pain, and renal disease, is more frequently associated with necrotic, crescentic, and fibrotic changes seen after biopsy. However, renal biopsy is rarely performed because of its invasive nature, and thus cannot be used to regularly assess disease activity.

In contrast to renal biopsy, several novel biomarkers have been identified in urine or blood by liquid biopsy due to recent progress in omics studies (Mucha et al. 2016). These biomarkers not only reflect pathological changes that have already occurred in the kidney but also reveal the pathophysiological processes underlying the disease. Thus, omics technologies contribute to the development of biomarkers that can be used for the diagnosis and monitoring of disease progression as another step towards personalised treatment.

The term omics refers to different techniques aiming to obtain molecular measurements of particles inside cells or tissue. Depending on the studied material they can be divided into genomics, epigenomics, proteomics etc. During the last decade, discoveries in omics studies have confirmed several hypotheses based on clinical observations; however, only genomics and proteomics have revealed the novel pathways involved in disease progression. Particularly noteworthy are studies on the role of complement; higher susceptibility to infection among IgAN patients; and comorbidities, particularly autoimmune diseases (Kiryluk et al. 2014).

Unfortunately, there is still no molecular diagnostic or prognostic biomarker derived from omic studies in IgAN that is widely used. Although some of the biomarkers, like circulatory Gd-IgA_1_ (Moldoveanu et al. 2007), Gd-IgA_1_ immune complexes with IgG (Tomana et al. 1997) or autoantibodies specific to Gd-IgA_1_ (Tomana et al. 1999) were validated in multiple studies, they have not been implemented in clinical settings for the disease diagnosis, prognosis or follow-up. However, the progress that has been made in basic science has helped to link omics with the immune response in IgAN. The crucial role of omics studies in providing data on the pathogenesis of IgAN has been recognised, resulting in the application of novel artificial intelligence and bioinformatic methods to data analyses. This underscores the importance of omics and confirms the viewpoint that omics studies will improve our understanding of IgAN in the future. Importantly, omics studies have already been applied to the research of other kidney diseases such as membranous nephropathy (Moszczuk et al. 2021).

In this review, we present the possible immunological link between omics studies and IgAN pathogenesis and extrapolate the multi-hit pathogenesis model to immune system dysregulation.

## Omics

### Genomics

Recent studies on IgAN suggest that it is a polygenetic disease with a high locus heterogeneity. In reported familial form of IgAN, the main pattern of inheritance is autosomal dominant with incomplete or variable penetrance (Magistroni et al. 2015). To date, several susceptibility loci for IgAN have been described in case–control genome-wide association studies (GWAS) (Gharavi et al. 2011; Kiryluk et al. 2014; Li et al. 2015; Yu et al. 2011). They indicate the crucial role of immunological dysregulation in IgAN pathogenesis with the involvement of antigen processing, complement activation, mucosal IgA production, innate immunity against pathogens, and maintenance of the intestinal barrier integrity. The correlation between local helminth diversity and the geographical prevalence of IgAN explain the higher genetic risk of IgAN in East Asia and favour the role of impaired intestinal immunity in the pathogenesis (Kiryluk et al. 2014). In the recent genome-wide association study Liu et al. (2022) described 20 significant loci, including 11 novel loci which were significantly associated with IgA serum levels and correlated positively with IgA nephropathy. Identification of novel genetic biomarkers of IgAN can also be achieved with the use of machine learning and statistic-based bioinformatic models. Such approach showed that five candidate genes (FOS, JUN, EGR1, FOSB, and DUSP1) can differentiate between IgA and healthy individuals, however, their biological role is yet to be described (Al Mehedi Hasan et al. 2022). The association between risk allele burden and the age of onset is suggestive of the cumulative effects of genetic variants on clinical disease characteristics (Kiryluk et al. 2014).

We hypothesise that dysfunction of the immune system at multiple levels is needed for disease progression. This may be supported by the fact that asymptomatic family members of IgAN patients have increased serum levels of IgA and IgA-IgG immunocomplexes (Miyazaki 1990; Suga 1985). GWAS loci explain approximately 6–8% of the disease risk (Magistroni et al. 2015). Most of the described loci are responsible for encoding well-known genes involved in immune processes, have pleiotropic effects, and can potentially be linked to the multi-hit pathogenesis model. The genes involved include human leukocyte antigen (HLA) genes, pro-inflammatory genes, complement activation genes, genes encoding crucial components of complement receptors, the B-cell activation and macrophage phagocytosis gene vav guanine nucleotide exchange factor 3 (*VAV3*), genes involved in the innate immunity mechanisms of mucosal membranes, the Ig class switching gene tumour necrosis factor superfamily member 13 (*TNFSF13*), and genes encoding interleukin (IL)-6-related cytokines and molecules involved in inflammatory diseases (Magistroni et al. 2015). Other genes related to IgAN susceptibility or increased serum Gd-IgA1 levels include the angiogenesis and cell proliferation gene 1-aminocyclopropane-1-carboxylate synthase-like protein 1 (*ACCS*), the gene encoding a glycosyltransferase involved in the generation of cell-surface determinants, differentiation antigens, and macrophage apoptosis regulator ST6 beta-galactoside alpha-2,6-sialyltransferase 1 (*ST6GAL1*), the transforming growth factor (TGF)-β pathway gene Kruppel like factor 10 (*KLF10*) (Li et al. 2015), and genes responsible for the enzymatic *0*-glycosylation of IgA1, which play a crucial role in aberrant IgA1 production (Gale et al. 2017; Kiryluk et al. 2017). Genomics can also be used for phenotype-genotype correlation in kidney biopsies of patients with glomerular diseases. In a small Polish study, it was suggested that *NR3C1* single nucleotide polymorphisms are associated with the clinical manifestations of IgAN and score in Oxford classification (Pac et al. 2021).

Genomic studies on IgAN have confirmed the multifaceted role of the immune system in its pathogenesis, which include the dysregulation of innate and adaptive immune responses. Moreover, studies have revealed the significant geospatial differences in susceptibility loci distribution and concomitance with other autoimmune diseases, indicating that different immune system disorders may lead to the same clinical manifestations described in IgAN. However, for most of GWAS loci, casual alleles still need to be defined by fine mapping and experimental follow-up studies.

### Epigenomics

Epigenomics refers to the study of reversible changes in DNA structure or histones and structural proteins that affect DNA availability and consequently gene expression but do not alter the DNA sequence. It remains the least explored omics field.

The molecular chaperone of core1β1, 3galactosyl transferase (Cosmc), has reduced expression in IgAN and is related to the production of Gd-IgA1. Interestingly, one epigenomic study showed that *Cosmc* expression in the B cells of paediatric patients with IgAN can be regulated by methylation of one of the promotor regions and affect the level of Gd-IgA1 (Sun et al. 2015). However, it was a small, targeted study on 50 (26 IgAN) patients and the results still need to be validated. One genome-wide screening for DNA methylation in 48 (24 IgAN) patients revealed two hypomethylated regions codifying genes involved in T-cell receptor (TCR) signalling, tripartite motif-containing 27, dual specificity phosphatase 3, and hypermethylation of vault RNA 2–1 (microRNA-886 [miR-886] precursor) (Sallustio et al. 2016). Hypermethylation of the miR-886 precursor results in decreased CD4^+^ T-cell proliferation after TCR signalling and increased expression of TGF-β. Together, these changes promote a shift in the proportion of T helper cells towards T helper type 1 (Th1) cells and a reduction in the TCR signal strength of CD4^+^ T cells. These results still need to be independently replicated in future studies.

Changes in miRNA expression are another form of epigenetic regulation that modulates gene expression in cells. miRNAs can serve as biomarkers and thus could be therapeutic targets in some immune-mediated diseases. In addition, the potential pathogenic role of several miRNAs has been found in immune cells. One study on 25 IgAN and 25 healthy individuals showed a correlation between the upregulation of miR-148b and downregulation of the core 1, β1,3-galactosyltransferase 1 (C1GALT1) enzyme (Serino et al. 2012). This impact on the enzyme responsible for IgA glycosylation may result in aberrant IgA production in IgAN. The results were then validated on an independent sample cohort of 50 IgAN and 50 healthy individuals in the same study. Another study on a group of 160 participants (72 IgAN), showed that miR let-7b, involved in the O-glycosylation of IgA by attachment of *N*-acetylgalactosamine to the hinge region, is upregulated in the peripheral blood mononuclear cells (PBMCs) of IgAN patients (Serino et al. 2015). Both results of miR-148b and let-7b were replicated and validated in a retrospective international study on 245 IgAN patients (Serino et al. 2016). MiR-374b, another miRNA targeting phosphatase and tensin homolog and Cosmc, thereby promoting B-cell proliferation and aberrant glycosylation of IgA (Hu et al. 2015) was shown to be overexpressed in a study of 30 IgAN and 15 healthy participants. miR-100-3p and miR-877-3p play roles in the stimulation of mesangial cells by Gd-IgA1 and may be involved in the immune processes damaging glomeruli, as was shown in the study of 76 IgAN patients (Liang et al. 2016). Their role in the pathogenesis still needs to be confirmed in the future. Several miRNAs have garnered interest as potential markers of renal fibrosis and glomerular sclerosis. miR-21-5p were associated with segmental glomerulosclerosis; and miR-21-5p, miR-214-3p, and miR-199a-5p were associated with interstitial fibrosis in a pilot study of 61 IgAN patients (Hennino et al. 2016). These results need to be confirmed in larger cohorts.

Apart from the potential diagnostic and monitoring value, epigenomics constitute an interesting source of targeted therapies, for instance aimed at the IgA_1_ O-glycosylation process. However, currently none of such therapies is available and the majority of epigenomic findings still needs to be replicated in independent studies.

### Transcriptomics

Transcription is a gene reading and rewriting process—a result of genetic and epigenetic information that has a great impact on defining the phenotype. Compared to genomics, transcriptomics shows the dynamics of a disease. Alterations in the two main pathways (WNT and PI3K/Akt) have been described in the PBMCs and B cells of 12 IgAN patients compared to 8 healthy controls (Cox et al. 2010). Both pathways have antiapoptotic and proliferative effects and enhance the proliferation of PBMCs. Moreover, the WNT pathway regulates B-cell proliferation through lymphoid enhancer factor 1. WNT pathway alterations are particularly seen in CD14^+^ CD16^+^ non-classical monocyte subsets with strong apoptosis potential. The study of 39 IgAN and 37 healthy individuals demonstrated differences in monocytes involving apoptosis signalling and mitochondrial dysfunction, and the expansion of CD14^+^ CD16^+^ monocytes in IgAN (Cox et al. 2015). Different expression of WNT and PI3K/Akt were also observed in other small-sample size studies of PBMCs and urine of IgAN patients (Liu et al. 2019; Luan et al. 2021; Wang et al. 2020). The dynamics of transcriptomics and IgAN pathophysiology are well demonstrated by upregulation of the immune-proteasome pathway and enhanced C-X3-C motif chemokine receptor 1 expression in PBMCs during macroscopic haematuria—a result of a whole-genome expression analysis in 12 IgAN patients (Cox et al. 2012). Transcriptomics can also be used for the analyses of pathological changes in kidney biopsies. IgAN glomeruli with endocapillary proliferation, compared to glomeruli without endocapillary proliferation, differentially express 424 genes encoding proteins involved in the innate immune response and classical complement pathway activation, M2 macrophage polarisation, and T-cell signalling pathways (Hodgin et al. 2014). This study of the transcriptome of 22 IgAN kidney biopsies followed the Connectivity Map approach elucidated pathways that could be potential targets of future therapies and that should be validated in the future studies. Moreover, the glomeruli and tubulointerstitial tissue of IgAN patients have different gene expression—Ebefors et al. (2011) in the study of 19 IgAN patients’ and healthy donors’ kidney biopsies showed that perlecan could become a prognostic marker, however, it still needs to be validated in larger cohorts. Some of the differentially expressed genes may serve as a molecular signature of proteinuria and potential biomarkers (Reich et al. 2010). Reich et al. (2010) showed in their study that 25 IgAN kidney biopsies could be distinguished from six control biopsies basing solely on the expression of 231 genes involved in albumin regulation and that the expression of an 11-transcript subset is related to the degree of proteinuria in IgAN and other glomerular diseases. We did not find any study of the expression of these genes in kidney biopsies replicating these results.

Recent years have brought dynamic development of transcriptomic techniques, of which single-cell RNA sequencing (sc-RNA-seq) is the most prominent example and which have also been applied to the study of IgAN pathogenesis. Sc-RNA-seq has enabled the discovery of novel immune mechanisms involved in the onset and progression of disease (Zheng et al. 2020). Zheng et al. (2020) demonstrated that complex gene expression alterations occur in mesangial cells, kidney-resident macrophages, CD8^+^ T cells, intercalated cells, and principal cells, leading to IgAN symptoms. The authors observed upregulation of the joining chain of multimeric IgA and IgM gene in mesangial cells. The product of this gene is necessary for Ig polymerisation and transport of IgA across the mucosal epithelium, which might play a role in IgA deposition in the mesangium. Other upregulated genes include several inflammation-related genes as well as WAP four-disulfide core domain 2, which promotes extracellular matrix accumulation. In the large-scale sc-RNA-seq study Chen et al. (2021) found high expression of Lymphotoxin Beta genes in kidney biopsies of IgAN and lupus nephritis patients—genes that could potentially be involved in B cells activation, increased expression of genes involved in interstitial fibrosis (tryptase beta 2 and tryptase alpha/beta-1) and finally high expression of CD47 in podocytes indicating a cross-talk between podocytes and macrophages.

In another sc-RNA-seq study, Tang et al. (2021) showed overexpression of novel genes involved in cell proliferation and matrix accumulation (metastasis-associated lung adenocarcinoma transcript 1, growth arrest, and DNA damage-inducible beta, SRY-box transcription factor 4 and EGF-like repeats and discoidin domains 3) in mesangial cells and overexpression of genes involved inflammatory pathways (TNF, IL-17 and NOD-like receptor signalling) in tubule cells. Moreover, the analysis of intracellular signalling suggested potential interactions between mesangial cells and other cells in IgAN. Although providing new, interesting insights into the molecular background of IgAN, number of sc-RNA-seq studies is limited and the available studies were based on small-sample sizes: Zheng et al. (2020)—19 patients (13 IgAN), Chen et al. (2021)—8 patients (3 IgAN), Tang et al. (2021)—5 patients (4 IgAN). More research with this technology applied in blood and kidney tissue is needed to provide better understanding of cell type-specific transcriptional states and responses in this disease.

Similar to genomics, transcriptomics studies have shown the complex involvement of the immune system in IgAN pathogenesis. Upcoming years will certainly provide new insights on the molecular background of IgAN thanks to emerging transcriptomic methodology such as RNA-seq.

### Proteomics

Proteomics, the study of polypeptide and protein patterns, is another promising approach for understanding the pathophysiology of IgAN and has provided some great discoveries. The unquestionable advantage of proteomic techniques is that they can be performed using urine samples, making it more convenient and less stressful for the patient and the physician. Below, we describe proteins of particular interest because of their role in the immune response.

A study of 30 IgAN Polish patients and 12 healthy individuals enabled the detection of 18 proteins potentially associated with the disease (Mucha et al. 2014), including complement system components, coagulation factors, extracellular matrix, intracellular and transmembrane proteins. Some of the results were also obtained in other studies (Guo et al. 2018; Kalantari et al. 2013; Moon et al. 2011; Prikryl et al. 2017). In another Polish study Krata et al. (2021) found different concentration of 2-Cys-peroxiredoxins, biomarkers of oxidative stress (Krata et al. 2018), in different glomerular pathologies, including IgAN and their association with lower glomerular filtration rates. C3 glomerular deposits are detected in most biopsies and complement system activation, which can be done by serum IgA (Hiemstra et al. 1987), causes glomerular damage via alternative and lectin pathways (Roos et al. 2006; Ohsawa et al. 2012). Oher proteins differentially expressed in the urine of IgAN patients were detected in a small study of 20 individuals (13 IgAN) and include alpha-1-antitrypsin—also obtained in other studies (Moon et al. 2011; Mucha et al. 2014; Prikryl et al. 2017; Surin et al. 2013); fibulin-5, which protects against oxidative stress; and osteopontin (Majd et al. 2018), which regulates immune responses, chemotaxis, nitric oxide production, and IL-17 production (Kaleta 2019). Some of these results need to be replicated in further studies. Alterations in heparin sulphate proteoglycans have been described in primary kidney diseases including IgAN and are thought to play a role in the inflammatory process, acting as chemoattractants for leukocytes (Celie et al. 2008). Among the four urinary proteins (intercellular adhesion molecule 1 [ICAM1], TIMP metallopeptidase inhibitor 1, serpin family C member 1 [SERPINC1], and adiponectin, C1Q and collagen domain containing [ADIPOQ]) identified as candidate biomarkers of IgAN in a study of the Uygur population (24 participants, including 12 IgAN), two may act as inflammation and oxidative stress modulators (ADIPOQ) or adhesion molecules important for the immune response (ICAM1) (Guo et al. 2018). SERPINC1 was also detected in the one study before (Mucha et al. 2014). Other described proteins with altered expression in IgAN include inflammation regulators such as uteroglobin and T-cell activators such as dipeptidyl peptidase four detected in the study of urine of 20 individuals (13 IgAN) which confirmed the results of previous studies (Samavat et al. 2015). In the recent study utilization of an enrichment-free one-pot sample preparation and ultra-high performance liquid chromatography-tandem mass spectrometry method identified 16 IgA1 O-glycopeptides as biomarkers of the disease (Chen et al. 2022). The proteomic findings are summarized in Table 1.

**Table 1:** Most significant urinary, proteomic findings with relation to the IgAN immunopathophysiology

References	Patients no. (total/IgAN)	No. of detected proteins	Most significant urinary proteins	Function/process
Moon et al. (2011)	30/12	216	Ceruloplasmin and α_1_ antitrypsin upregulation Aminopeptidase N and vasorin precursor downregulation	Coagulation, TGF-β/growth factors inhibition, cell adhesion
Surin et al. (2013)	73/43	4	α_1_—β-glycoprotein precursor and α_1_ –antitrypsin precursor upregulation LG3 fragment of endorepellin downregulation	Inhibition of neutrophil elastase attack, complement activation and regulation, coagulation, inhibition of angiogenesis
Rocchetti et al. (2013)	167/63	13	Free κ light chains and perlecan LG3 downregulation	Chronic inflammatory state in chronic kidney disease, inhibition of angiogenesis
Kalantari et al. (2013)	13/13	18	α_1_-1-microglobulin, hemopexin, apolipoprotein A-I, complement C3, vitamin D-binding protein, β-2-microglobulin, retinol-binding protein 4 upregulation	Extra cellular matrix-receptor interaction, Complement activation, coagulation
Mucha et al. (2014)	60/30	18	Ceruloplasmin, complement 3, complement C4-A, α_1_—antitrypsin, α_1_-2-macroglobulin, apolipoprotein A-1, antithrombin-III, haptoblobin, prothrombin	Complement activation, coagulation
Samavat et al. (2015)	20/13	46	Uprotocadherin-1, uteroglobin, dipeptidyl peptidase 4 upregulation CD44, apolipoprotein D, phosphoinositide-3-kinase-interacting protein 1, pancreatic secretory granule membrane major glycoprotein 2, vasorin, poliovirus receptor, epidermal growth factor downregulation	Antiapoptotic effects, response to reactive oxygen species Antigen transcytosis by M cells, Cell adhesion, T-cell mediated cytotoxicity, T-cell activation, TGF-β/growth factors inhibition
Prikryl et al. (2017)	40/20	30	α-1-antitrypsin, apolipoprotein A-1 upregulation Kininogen downregulation	Inhibition of neutrophil elastase attack, Response to reactive oxygen species
Guo et al. (2018)	24/12	277	ADIPOQ, SERPINC1, ICAM1, TIMP1 upregulation	Modulation of inflammation and oxidative stress, Coagulation, Extra cellular matrix regulation
Taylor et al. (2019)	32/16	325	Phosphatidylethanolamine binding protein- 4, lysosomal and proteolytic proteins upregulation	Protease inhibitor, B-cell activation (unknown)
Fang et al. (2021)	52/19	276	α_1_ β-glycoprotein and afamin upregulation	Complement activation and regulation, coagulation

Data generated by proteomics studies in IgAN are complex and difficult to analyse. The role of the described proteins in pathogenesis should be interpreted cautiously as most are biomarkers of the disease and not causative factors. In the future, application of artificial intelligence and machine learning may significantly improve the analyses of proteomics studies and result in discoveries leading to understanding IgAN pathogenesis.

### Multi-Omics

Multi-omics is the most comprehensive approach used for the study of background of a disease. It combines multiple techniques including genomics, epigenomics, transcriptomics, and proteomics. This combination results in a holistic image depicting interlinks between molecular findings of each method and explains many pathophysiological traits involved in pathogenesis. It helps in the search for associations between omic data and phenotype. A subset of mult-omics called single-cell multi-omics provides an unprecedent resolution to look at functional and pathophysiological properties of a single cell in health and disease. The multi-omic approach helped to determine changes induced by miR-223 in monocytes/macrophages (M’baya-Moutoula et al. 2018) and this technique could be analogically applied in IgAN.

Mutli-omic approaches can also enhance interpretation of GWAS loci. Because many of GWAS loci reside in non-coding regions, combination of cell type-specific epigenetic and transcriptomic profiling enables mapping of GWAS signal to specific cell types and specific transcripts using computational prioritization strategies (Cano-Gamez and Trynka 2020). These problems can be addressed with novel methods integrating GWAS data with cell type-specific date including single nucleotide polymorphisms (SNPs) enrichment approaches, colocalizations analysis integrating GWAS and expression quantitative trait loci (eQTL), and transcriptome-wide association studies (Cano-Gamez and Trynka 2020; Li and Ritchie 2021). They enable identification of cell types and tissues involved in a disease, targeting pathogenic genes and association of genes with transcriptome and phenotype, respectively. Such combined methods enabled recognition of the involved cells in lupus erythematosus and rheumatoid arthritis (Hu et al. 2011), identification of loci regulating low-density lipoprotein levels (Musunuru et al. 2010) or confirmed and explained association of a gene with type 1 diabetes (Gamazon et al. 2015).

One of the combined mutli-omic approaches has been successfully applied also in IgAN. Gillies et al. (2018) identified significant genes in microdissected glomeruli and tubulointerstitial transcriptomes by integrating GWAS and eQTL data to perform TWAS. They primarily considered the HLA region, especially decreased *HLA-DRB5* and increased *HLA-DPA1* and *HLA-DPB1* mRNAs expression in glomeruli and tubulointerstitial tissue (Gillies et al. 2018). Another example of a combined approach including also meta-analysis of consolidated data from -omic studies and bioinformatic methods is the study by Krochmal et al (2017). Through analysis of available urinary proteomic studies, pathway identification, analysis of transcriptomic database (Nephroseq), functional protein evaluation and literature mining IgAN-relevant targets (3/232 proteins) were chosen and validated with the immunohistochemistry (IHC) of kidney tissue. This proves a successful exploitation of integrative -omic data and bioinformatic analysis which lead to in biologically relevant results, as three of proteins (adenylyl cyclase-associated protein 1, SHC-transforming protein 1 and prolylcarboxypeptidase) were showed to be overexpressed in IgAN in comparison to healthy control group, but not to other glomerular diseases (Krochmal et al. 2017). This study provides a shape of how -omic findings can be used in clinical setting, however, its limitation was small-sample size used for IHC (8 IgAN patients). A liquid chromatography-tandem mass spectrometry (LC–MS/MS) enables combined approach for metabolomics and proteomics. In the recent study LC–MS/MS based approach revealed new molecular insights and identified candidate biomarkers—a protein kinase involved in neuroinflammation (PRKAR2A), a subunit of IL-6 receptor, a guanine nucleotide exchange factor (SOS-1) and palmitoleic acid (Zhang et al. 2022).

Altogether, combined multi-omic approach and extensive bioinformatic analysis of available databases (ex. Nephroseq) is a natural direction for molecular studies in IgAN as it provides a full image of pathophysiology.

### New Therapeutic Approaches Involving Nanotechnology and Gene Editing

Omic findings enable identification of innovative diagnostic and prognostic biomarkers as well as new treatment targets in kidney disorders. However, to benefit from these discoveries simultaneous development of new methods enabling biomarker detection or targeted drug delivery is needed. In the future it could be achieved thanks to the nanotechnology. This term refers to the engineering of atoms and molecules at the submicron scale (Kamaly et al. 2016). Such modification of nanoparticles (NPs) can lead to the improved pharmacokinetics and biodistribution of the drugs they carry. It can result in a more specific, targeted action of an agent and a reduction in drug toxicity and systemic effects. Such technologies have already been applied in some experimental studies of kidney disorders including, glomerular diseases and IgAN (Kamaly et al. 2016). In a mouse model of IgAN treatment with a liposome loaded with prednisolone phosphate reduced IgA and C3 glomerular deposits to a higher degree than a prednisolone alone (Liao et al. 2001). Low-molecular-weight NPs have been showed to successfully deliver small interfering RNA (siRNA) to endothelium, including kidney tissue, and silence multiple endothelial genes (Dahlman et al. 2014). Another study in mice showed that polycationic cyclodextrin NPs can specifically deliver siRNA to mesangial cells with limited deposition in other areas of kidney (Zuckerman et al. 2015). Such strategies could be used for the delivery of future therapeutics like siRNA, mRNA or DNA inhibitors derived from omic studies directly to the glomerulus, but still need further examination and clinical studies.

The use of clustered regularly interspaced short palindromic repeats (CRISPR)/CRISPR-associated (Cas) systems (Jinek et al. 2012) is another promising approach that could support application of novel omic findings. which is presently used only in preclinical studies. CRISPR/Cas9 helped to the determine the role of apoptosis inhibitor of macrophage (AIM) protein in IgA deposition-associated glomerular injury in mice. AIM-deficient IgAN model although possessed IgA deposits similar to the wild-type, it did not exhibit glomerular accumulation of IgM/IgG complements, CD45^+^ leukocytes infiltration and upregulation of inflammatory and fibrogenic genes, which occurred only after AIM administration (Takahata et al. 2020). This demonstrates that CRISPR/Cas9 could be used for functional assessment of omic findings and in the future potentially used as a treatment method, however, it still needs further preclinical and then clinical examination.

## Multi-Hit Pathogenesis Model

**Fig. 1:**
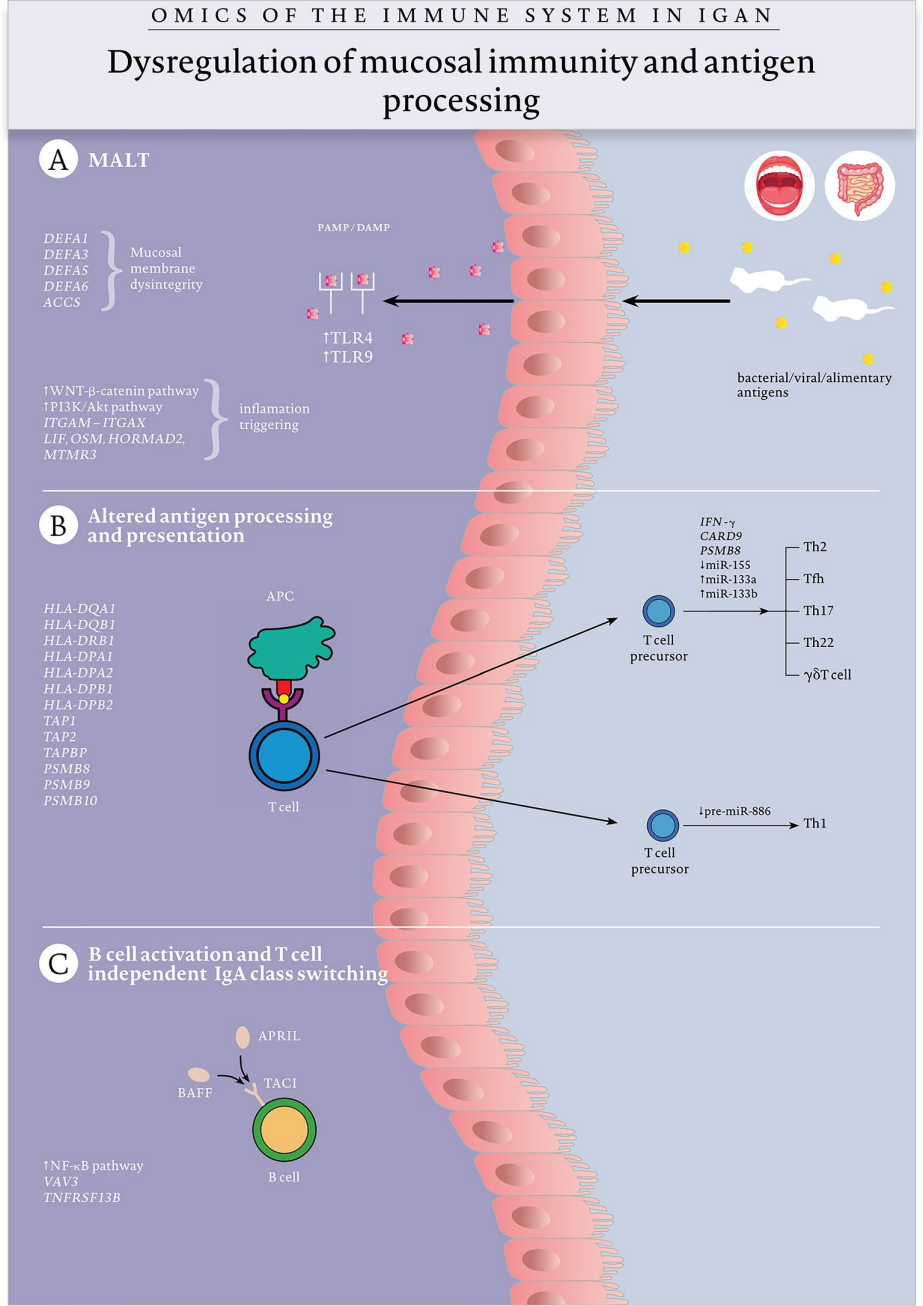
Omics and mucosal immunity dysregulation in IgA nephropathy (hit 1). Dysregulation of mucosal immunity proceeds at various sites and involves both innate and adaptive mechanisms. **A** The aberrant response at MALT in the intestine and upper respiratory tract is triggered by bacterial, viral, and alimentary antigens. Risk variants of IgAN: *DEFA1*, *DEFA3*, *DEFA5, DEFA6, ACCS* are probably associated with lack of integrity of the mucosal membrane, promoting an excessive immune response. Overexpression of TLR4 and TLR9 enhances the recognition of pathogen-associated molecular pattern (PAMP) and damage-associated molecular pattern (DAMP), respectively, triggering a first-line innate immune response to the antigens. The innate response and inflammation are provoked and mediated via two pathways hyperactivated in IgAN: WNT-β-catenin and PI3K/Akt. Additionally, several IgAN risk variants: *ITGAM-ITGAX*, *LIF*, *OSM*, *HORMAD2* and *MTMR3* are thought to play a role in inflammation and involvement of both innate and adaptive immunity. **B** *HLA-DP*, *HLA-DQ, HLA-DR*, *TAP* and *PSMB* are risk variants of genes crucial for antigen processing and presentation—a process that takes place at the MALT and is a bridge between innate and adaptive responses. Antigen presentation and stimulation of T-cell by antibody-presenting cells (APCs) leads to T-cell activation, proliferation and differentiation. One theory, supported by upregulation of miR133a and miR133b, downregulation of miR-155, the existence of risk variants: *IFN-*γ, *CARD9* and *PSMB8*, assumes a shift towards Th2, T follicular helper, Th17, Th22, and gamma delta T cells. The second, supported by the downregulation of pre-miR-886, assumes an imbalance towards Th1 cells. **C** APRIL and BAFF, overexpressed in IgAN, are key molecules in B-cell activation and T-cell-independent IgA class switching; B-cell activation is also mediated via the nuclear factor kappa B pathway, which is upregulated in IgAN, and expression of the IgAN risk alleles *vav guanine nucleotide exchange factor 3 (VAV3)* and *TNFRSF13B*

**Fig. 2:**
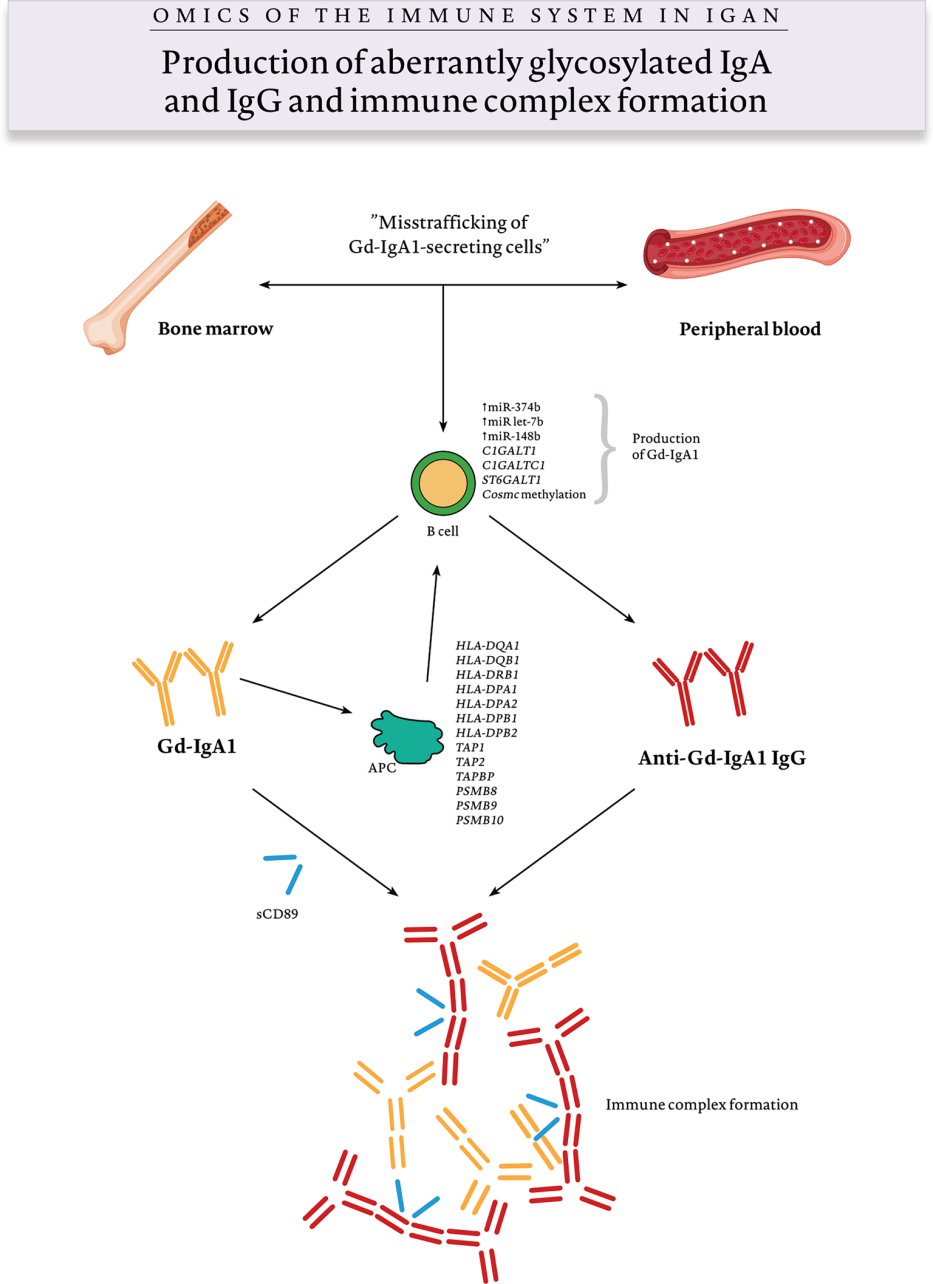
Omics and production of aberrantly glycosylated IgA, anti-IgA IgG, and immune complex formation (hits 1, 2, 3). Dysregulation of mucosal immunity coexists with the production of aberrantly glycosylated, Gd-IgA1 by overstimulated B cells. Gd-IgA1 reaches the circulatory system most likely via “misstrafficking” of plasma cells to the bone marrow. Aberrant glycosylation is a result of overexpression of miR-374b, miR let-7b, miR-148b; IgAN risk variants are associated with IgA production: *C1GALT1*, *C1GALTC1*, *ST6GALT1*, and methylation of *Cosmc*. IgAN risk variants of genes involved in antigen processing and presentation affect anti-Gd-IgA1 IgG production and along with the overexpression of sCD89 are crucial for immune complex formation

**Fig. 3:**
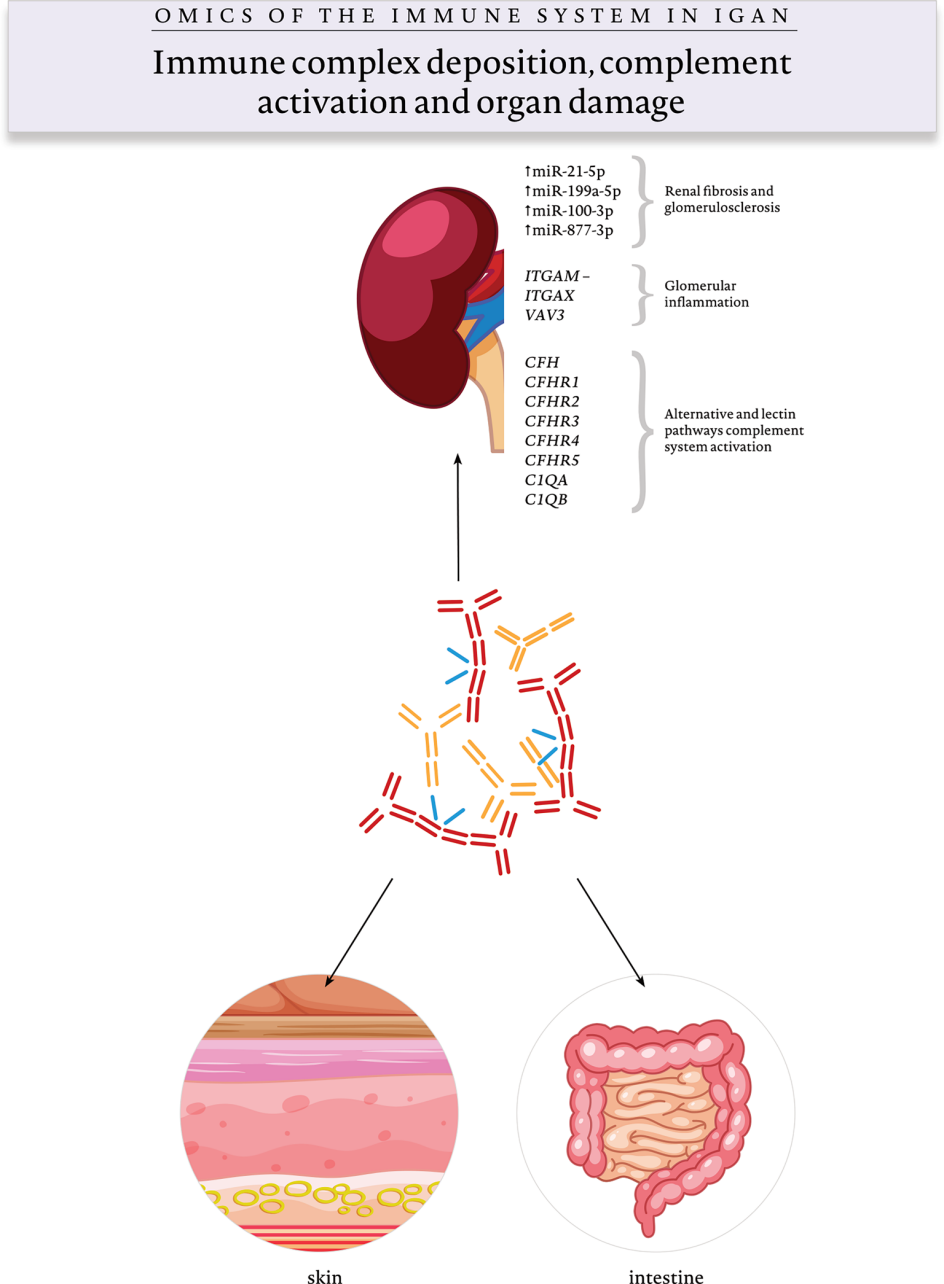
Omics and immune complex deposition, complement activation, and organ damage (hit 4). Renal and organ damage in IgAN is mediated via complement system activation, of which several risk variants have been described including *CFH*, *CFHR*, and *C1Q* gene groups. IgAN risk variants, *ITGAM-ITGAX* and *VAV3*, are thought to play a role in glomerular inflammation and mesangial proliferation, as well as the upregulation of miR-1-5p, miR-199a-5p, miR-100-3p, and miR-877-3p in fibrosis and glomeruli sclerosis. For unknown reasons, immune complexes in some IgAN variants are also deposited in the intestine and skin

The current pathogenesis model divides the development of IgAN into four steps – so-called “hits” (Suzuki et al. 2011). The originally proposed hit 1 assumes there is increased ability to produce aberrantly glycosylated, circulatory Gd-IgA1 in affected individuals in response to bacterial, viral, or alimentary antigens (Fig. 1). The affected glycosylation concerns galactose deficiency in the hinge region of IgA1. Gd-IgA1 is either a product of “misstrafficking” of overly stimulated mucosal plasma cells to the bone marrow or a result of hyperstimulation of the mucosal immune system and “spill-over” from mucosal membranes. The potential function of Gd-IgA1 is unknown; however, recent GWAS studies have shown a correlation between local helminth diversity and frequency of risk alleles. It remains to be seen if some of the alleles could be involved in Gd-IgA1 secretion, suggesting that an enhanced IgA response could be protective against worm infection and may explain the West-to-East gradient in incidence and exacerbation of symptoms during upper respiratory tract infections. In hit 2, Gd-IgA1 is recognised by the adaptive immune system and triggers the production of circulatory IgG necessary for hit 3—immune complex (IC) formation (Fig. 2). Complexes are thought to be formed after interaction with the soluble cluster of differentiation 89 (sCD89) receptor, the levels of which can predict disease recurrence (Berthelot et al. 2015). In hit 4, ICs are deposited in the glomerular mesangium of the kidneys and cause a local inflammatory process, mesangial cell activation, glomerulosclerosis, fibrosis, and renal injury (Fig. 3). The reason that ICs are deposited in the kidneys is unknown. Kidneys seem to be “innocent bystanders”—victims of immune system dysregulation. In IgA vasculitis, an IgAN variant, IC deposits have been described in other sites including the intestine or skin.

Although the multi-hit model aptly identifies key steps in IgAN development and is, to date, the best description of IgAN pathogenesis, it is still in development to fully depict the complexity of immune system dysregulation. It should recognize that immune system dysregulation may be involved at different points (e.g., complement system activation may play a role in mucosal immunity dysfunction, as well as in glomerular damage).

## Treatment

The Kidney Disease Improving Global Outcomes guidelines describe two therapeutic options. The first one is based on non-immunosuppressive treatment with angiotensin-converting enzyme inhibitors or angiotensin II receptor blockers and is used in all patients with proteinuria > 0.5 g/24 h irrespective of if they have hypertension. The second one assumes the use of corticosteroids for six months in patients who remain at high risk of progressive chronic kidney disease despite three months of maximal supportive care defined as proteinuria > 1 g/24 h. However, in light of recent studies (Lv et al. 2017; Rauen et al. 2015), such therapy should be administered cautiously, and its benefits are not well established in a selected groups of patients including those with estimated glomerular filtration rate < 30 mL/min/1.73 m^2^, obesity, or diabetes. Nevertheless, none of these approaches target the root cause of the disease and should be considered a symptomatic treatment. Moreover, steroid therapy in some patients is ineffective, suggesting different defects in the immune system among IgAN individuals. An understanding of the overall changes that occur in the immune system in IgAN may facilitate the development of an effective targeted therapy.

## Omics, the Immune System, and IgAN Pathogenesis

The immunopathogenic mechanisms that lead to the development of IgAN are complex and involve the engagement of both adaptive and innate immunity. IgAN is a multifactorial disease and there is no single impairment or defect in the immune system that results in disease onset. In Figs. 1, 2, and 3, we show the latest omics findings underlying immune system dysregulation with respect to the multi-hit pathogenesis model.

### Mucosal Immunity

Haematuria and proteinuria in IgAN are exacerbated during upper respiratory tract infections, suggesting an exaggerated immune response to pathogens in the mucosa-associated lymphoid tissue (MALT) of the respiratory tract (Fig. 1a). In healthy individuals, MALT is the site of production of IgA by plasma cells originating from immune-competent B cells after migration to the lamina propria. They secrete dimeric and polymeric IgA1 and IgA2, of which the vast majority reach the mucosal fluids. The mucosal production of IgA can also be the result of a T cell independent mechanism involving secretion of IL-6, IL-10, TGF-β, B-cell activating factor (BAFF), and a proliferation-inducing ligand (APRIL) (Fig. 1c). A monomeric circulatory form of IgA1 is also produced in the bone marrow, and together with its CD89 receptor, is thought to play a regulatory role in inflammation. IgA found in the sera of IgAN patients is aberrantly glycosylated in the hinge region and can either originate from the bone marrow as an effect of overexpression of the T-cell homing receptor (Batra et al. 2007) or mucosal sites as the effect of an exaggerated immune response in MALT and “spill-over” to the circulatory system (Fig. 2). Moreover, MALT is the site of the production of antimicrobial peptides such as defensins, which are involved in innate immunity and the response to pathogens.

The aberrations in the mucosal immune response have been confirmed in a study by Coppo (2015) which has indicated that gut-associated lymphoid tissue (GALT) is even more crucial for pathogenesis. Dysregulation of GALT, the largest mass of lymphoid tissue in the body, is thought to lead to defective responses to microbiota and alimentary antigens, resulting in Gd-IgA1 production (Fig. 1a). Overstimulation by bacterial lipopolysaccharides (LPS) may lead to abnormal IgA glycosylation (Qin et al. 2008). Moreover, intestinal inflammation manifests as an increase in intestinal CD3^+^ cells, and cyclooxygenase 2-positive cells have been found in IgAN patients; the degree of inflammation is correlated with the degree of serum IgA, proteinuria, and hematuria (Honkanen et al. 2005).

Omics results have pointed to the important role of mucosal immunity in the pathogenesis of IgAN. GWAS have identified several candidate defective genes in IgAN that play a role in the mucosal response including: *TNSF13* (Kiryluk et al. 2014) encoding APRIL, a B-cell-stimulating cytokine responsible for T-cell-independent IgA class switching induced by intestinal bacteria; *leukaemia inhibitory factor* and *oncostatin M* (Gharavi et al. 2011) encoding IL-6-related cytokines that have a role in mucosal inflammation; *integrin alpha M-integrin alpha X* encoding an integrin responsible for leukocyte migration, cell adhesion, and phagocytosis by macrophages (Kiryluk et al. 2014); and *defensin alpha* (Yu et. al. 2011) encoding an alpha-defensin produced in MALT. The complexity of the dysregulation of mucosal immunity and its consequences are shown in Fig. 1.

### Innate Immunity

As described above, a crucial component of innate immunity is MALT. Dysregulation at this site involves several key particles and cells, which are crucial for dysregulation of innate immunity at mucosal sites.

Toll-like receptors (TLRs) are responsible for the recognition of pathogen-associated molecular patterns and damage-associated molecular patterns (Fig. 1a). Their expression affects the immune response and is associated with severe disorders. For example, in liver diseases, impaired TLR expression leads to inflammation, fibrogenesis, and liver injury (Żeromski et al. 2020), and omics studies have shown their impaired function in IgAN. Polymorphisms in *TLR9* are suspected to play a role in murine and human IgAN, leading to Th1 polarisation and renal injury (Suzuki et al. 2008). Moreover, the expression of *TLR9* is correlated with the efficacy of treatment (Sato et al. 2012), and low *TLR9* copy number and its mRNA expression are correlated with renal function in IgAN patients (Sallustio et al. 2015). TLR4 is overexpressed in mononuclear cells, which may play a role in impairment of the innate immune response (Coppo et al. 2010). Physiologically, it is activated by LPS. TLR4 signalling and expression may also cause mesangial cell activation and renal damage due to pro-inflammatory cytokine secretion (Lim et al. 2011). However, the findings about TLR involvement have not been replicated in different populations and were not confirmed by GWAS studies (Kiryluk et al. 2014; Li et al. 2015; Yu et al. 2011).

GWAS findings are suggestive of a susceptibility role of *DEFA* genes in the IgAN (Kiryluk et al. 2014; Li et al. 2015; Yu et al. 2011). They are responsible for encoding antimicrobial particles, that act as endogenous antibiotics and constitute important part of mucosal immunity and inflammatory response to infection (Ganz 2003). Alpha-defensins chemoattract naïve T cells, immature dendritic cells and monocytes and induce release of IL-8, monocyte chemoattractant MCP-1—particles increased in urine of IgAN patients (Stangou et al. 2009; Yokoyama et al. 1998).

Another interesting innate immunity finding coming from GWAS study is a susceptibility role of *CARD9* encoding caspase recruitment domain-containing protein 9 (CARD9)—a protein promoting nuclear factor-κB in macrophages (Kiryluk et al. 2014). One of the CARD9 SNPs has been shown to be associated with increased CARD9 expression and increased risk of ulcerative colitis and Crohn’s disease (Franke et al. 2010; McGovern et al. 2010). In mice CARD9 was involved in intestinal repair, Th17 responses and control of intestinal epithelial injury (Sokol et al. 2013). CARD9 constitutes an interesting molecule with referral to hypothesis about intestinal-renal connection in IgAN (Coppo 2015), that should be functionally evaluated in the future.

Complement system activation is suspected to be one of the main drivers of kidney injury in IgAN (Fig. 3). The presence of C3 and C4d deposits in mesangial cells in the absence of C1q indicates alternative and lectin pathway activation. C3 deposits correlate with disease severity and progression (Kim et al. 2012). The changes in plasma C3 activation products indicate that the complement system has a pathogenic role not only locally in kidneys but also systemically. Renal injury may be the result of mesangial cell activation by Gd-IgA1 via an alternative pathway, which is enhanced by overexpression of the mesangial IgA receptor CD71 and interaction of IgA with ficolins, leading to lectin pathway-mediated injury (Tortajada et al. 2019). Proteomics have confirmed the presence of C3 breakdown products in IgAN (Knoppova et al. 2016), and recent genomic studies have revealed the association between certain single nucleotide polymorphisms and copy number variants of complement-related loci and IgAN (Gharavi et al. 2011; Kiryluk et al. 2014). Figure 3 presents the omics behind complement system activation and renal damage.

### Adaptive Immunity

The alterations in adaptive immunity in IgAN extend far beyond IgA production or B-cell function and also involve T-cell signalling and antigen presentation—a bridge between innate and adaptive immunity.

As described above, omics have revealed the molecular mechanisms underlying the secretion of Gd-IgA1. They include two potentially significant loci (*core 1 synthase, glycoprotein-N-acetylgalactosamine 3-beta-galactosyltransferase 1* [*C1GALT1*] and *C1GALTC1*), which may be involved in defective IgA glycosylation in IgAN patients and should be considered potential therapy targets. Expression of *C1GALT1* is regulated by miR-148b, another molecule upregulated in IgAN. Another omics finding involved in IgA production is the upregulation of miR let-7b and miR-374b (Fig. 2).

Antigen presentation, a process essential to antibody production, seems to be engaged at several points during the IgAN pathogenesis. GWAS studies have highlighted variants of several risk alleles that are involved in antigen processing and presentation including *transporter 1, ATP binding cassette subfamily B member* (*TAP1*) and *TAP2*, which are responsible for antigen binding to major histocompatibility complex (MHC) class I and MHC class II loci—*HLA-DQA1*, *HLA-DQB1*, *HLA-DRB1*, and *HLA-DP*. These loci may have a role in the dysregulation of the intestinal IgA production (Li et al. 2014) and anti-IgA1 IgG antibody production because of their permissive character in autoimmunity (Magistroni et al. 2015). Transcriptomic studies have revealed the altered expression of proteasome 20S subunit beta 8 (*PSMB8*)*, PSMB9*, *PSMB10*, and *TAP binding protein* genes responsible for upregulation of the immunoproteasome pathway in IgAN patients, which is crucial for MHC class I antigen presentation (Schena et al. 2018). The omics of Gd-IgA1, anti-Gd-IgA IgG production, and immune complexes formation is shown in Fig. 2.

The most prominent alterations of B cells described in IgAN, apart from defects in IgA glycosylation, are the aberrant expression of APRIL and BAFF. Their main roles are B-cell proliferation, maturation and promotion of T-cell-independent IgA class switching. Elevation of APRIL expression in the B cells of IgAN patients has been described and promotes the hypersecretion of Gd-IgA1 (Zhai et al. 2016). Overexpression of APRIL has also been shown in IgAN tonsillar germinal centres (Muto et al. 2017). Elevated levels of BAFF lead to Gd-IgA1 overexpression and are associated with renal function in IgAN (Li et al. 2014). Another IgAN omics change includes the upregulation of miR-374b, which promotes B-cell proliferation and aberrant IgA glycosylation.

The role of T cells in IgAN is not fully understood; however, they appear to be hyperactivated and have an altered proportion of subsets. In a small study of primary glomerulonephritides with 12 IgAN patients, the lower expression of cytotoxic T lymphocyte-associated antigen 4 (CTLA4) on the membrane of T and B cells was described (Grywalska et al. 2019). The lower expression of CTLA4 indicates activation of a higher number of T cells and an increased concentration of pro-inflammatory cytokines. In this study, an inverse correlation between CTLA4 expression and kidney function and serum Ig concentration was found. Studies on the distribution of T-cell subsets have shown contradictory results. Most of them have indicated higher proportions of circulatory Th2, T follicular helper, Th17, Th22, and gammadelta T cells (Ruszkowski et al. 2019) (Fig. 1b). These findings are supported by omics studies. One interferon (IFN)-γ polymorphism has been described as a risk allele of IgAN (Schena et al. 2006). IFN-γ promotes a shift toward Th1 cells, and its high expression might have a protective role. Another IgAN risk loci, *caspase recruitment domain family member 9* (*CARD9*) and *PSMB8*, encode proteins necessary for Th17 differentiation. Similarly, some epigenomic changes provide information on T-cell involvement. Downregulation of miR-155 in PBMCs, Th2 and Th17 shift (Yang et al. 2017), and upregulation of miR-133a and miR-133b in PBMCs have been observed in IgAN patients (Jin et al. 2018). Interestingly, the study of miR-886 is contradictory to the studies described above. Hypermethylation of its precursor leads to decreased CD4^+^ T-cell proliferation and an imbalance towards the Th1 subset (Fig. 1b).

Omics studies have already revealed several defects in the immune system of IgAN patients; however, we still do not fully understand the immunopathology behind the disease, for example, why certain individuals have increased Gd-IgA1 levels and do not develop kidney disease, where the production of Gd-IgA1 occurs, and why immunocomplexes deposit in the kidney and how they cause damage. Future omics studies are needed to address these questions.

## Conclusions

As described, omics studies have suggested impairment of both innate and adaptive immunity as a cause of IgAN. We propose a more holistic approach to the multi-hit pathogenesis model to emphasise the multifaceted immunological changes in IgAN and its heterogeneity. It remains unknown whether there are any primary changes that may influence the occurrence of other immune disorders in IgAN and trigger the disease. The attenuation of IgAN after bone marrow transplantation in patients and murine models (Hoshino et al. 2014; Imasawa et al. 1999; Park et al. 2008) indicates the existence of such primary changes and their location in stem cells. Future omics studies are needed to provide a better understanding of IgAN pathogenesis and to identify specific biomarkers, thus facilitating the development of individualised and safer therapy.

## Data Availability

Not applicable.
